# Systematic review assessing the effectiveness of dietary intervention on gut microbiota in adults with type 2 diabetes

**DOI:** 10.1007/s00125-018-4632-0

**Published:** 2018-05-12

**Authors:** David Houghton, Timothy Hardy, Christopher Stewart, Linda Errington, Christopher P. Day, Michael I. Trenell, Leah Avery

**Affiliations:** 1 Institute of Cellular Medicine, 4th Floor William Leech Building, Newcastle University, Newcastle upon Tyne, NE2 4HH UK; 2 Liver Unit, Freeman Hospital, Newcastle Upon Tyne Hospitals NHS Trust, Newcastle Upon Tyne, UK; 3 Department of Molecular Virology and Microbiology, Baylor College of Medicine, Houston, TX USA; 4 Faculty of Medical Sciences, Newcastle University, Newcastle upon Tyne, UK

**Keywords:** Diet, Exercise, Gut microbiota, Intervention, Physical activity, Systematic review, Type 2 diabetes

## Abstract

**Aims/hypothesis:**

Despite improved understanding of the pathophysiology of type 2 diabetes mellitus, explanations for individual variability in disease progression and response to treatment are incomplete. The gut microbiota has been linked to the pathophysiology of type 2 diabetes mellitus and may account for this variability. We conducted a systematic review to assess the effectiveness of dietary and physical activity/exercise interventions in modulating the gut microbiota and improving glucose control in adults with type 2 diabetes mellitus.

**Methods:**

A systematic search was conducted to identify studies reporting on the effect of dietary and physical activity/exercise interventions on the gut microbiota and glucose control in individuals with a confirmed diagnosis of type 2 diabetes mellitus. Study characteristics, methodological quality and details relating to interventions were captured using a data-extraction form. Meta-analyses were conducted where sufficient data were available, and other results were reported narratively.

**Results:**

Eight studies met the eligibility criteria of the systematic review. No studies were found that reported on the effects of physical activity/exercise on the gut microbiota and glucose control. However, studies reporting on dietary interventions showed that such interventions were associated with modifications to the composition and diversity of the gut microbiota. There was a statistically significant improvement in HbA_1c_ (standardised mean difference [SMD] −2.31 mmol/mol [95% CI −2.76, −1.85] [0.21%; 95% CI −0.26, −0.16]; *I*^2^ = 0%, *p* < 0.01), but not in fasting blood glucose (SMD −0.25 mmol/l [95% CI −0.85, 0.35], *I*^2^ = 87%, *p* > 0.05), fasting insulin (SMD −1.82 pmol/l [95% CI −7.23, 3.60], *I*^2^ = 54%, *p* > 0.05) or HOMA-IR (SMD −0.15 [95% CI −0.63, 0.32], *I*^2^ = 69%, *p* > 0.05) when comparing dietary interventions with comparator groups. There were no significant changes in the relative abundance of bacteria in the genera *Bifidobacterium* (SMD 1.29% [95% CI −4.45, 7.03], *I*^2^ = 33%, *p* > 0.05), *Roseburia* (SMD −0.85% [95% CI −2.91, 1.21], *I*^2^ = 79%, *p* > 0.05) or *Lactobacillus* (SMD 0.04% [95% CI −0.01, 0.09], *I*^2^ = 0%, *p* > 0.05) when comparing dietary interventions with comparator groups. There were, however, other significant changes in the gut microbiota, including changes at various taxonomic levels, including phylum, family, genus and species, Firmicutes:Bacteroidetes ratios and changes in diversity matrices (α and β). Dietary intervention had minimal or no effect on inflammation, short-chain fatty acids or anthropometrics.

**Conclusions/interpretation:**

Dietary intervention was found to modulate the gut microbiota and improve glucose control in individuals with type 2 diabetes. Although the results of the included studies are encouraging, this review highlights the need for further well-conducted interventional studies to inform the clinical use of dietary interventions targeting the gut microbiota.

**Electronic supplementary material:**

The online version of this article (10.1007/s00125-018-4632-0) contains peer-reviewed but unedited supplementary material, which is available to authorised users.

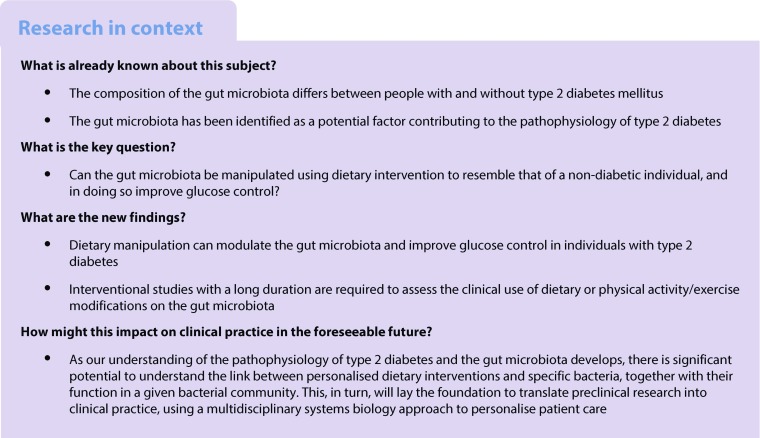



## Introduction

The incidence of type 2 diabetes mellitus is steadily increasing and the worldwide incidence is predicted to exceed 500 million by 2030 [1]. Although pharmacotherapy offers a management option for individuals with type 2 diabetes, lifestyle interventions, including modifications to diet and physical activity/exercise levels with weight loss of 5–10%, remain the cornerstone of treatment [2]. Nonetheless, concerns remain relating to adherence, implementation, specificity and a lack of long-term randomised trials in clinical practice, and these hinder the clinical use of such interventions [3]. Furthermore, there is substantial inter-individual variability in disease pathophysiology and response to treatments [4].

A potentially new therapeutic target receiving considerable interest is the collection of microorganisms, specifically bacteria that reside within the gastrointestinal tract, which are termed the ‘gut microbiota’. Advances in molecular sequencing and computational methods have provided an unprecedented understanding of how the gut microbiota functions in a symbiotic nature with the host, contributing to nutrition, metabolism, immune response and intestinal architecture [5, 6]. Alterations in the composition of the gut microbiota, termed ‘dysbiosis’ [7], have been linked to conditions including type 2 diabetes mellitus [8, 9] and metabolic disorders [10, 11]. Although the exact mechanisms linking the gut microbiota and type 2 diabetes mellitus remain unknown, differences in the composition of the gut microbiota may contribute to the variability observed among individuals with type 2 diabetes mellitus [12].

The gut microbiota influences energy harvest [12], blood glucose [13] and the effectiveness of pharmacotherapy [14]. Furthermore, targeting the gut microbiota with lifestyle interventions has been shown to result in significant changes in bacterial composition that are aligned with improvements in glucose control [15–25]. However, studies to date have generally been in animal models or in individuals without a clinical diagnosis of type 2 diabetes mellitus. Our aim was to conduct a systematic review to identify studies of lifestyle interventions (i.e. dietary and physical activity/exercise interventions) involving adults with type 2 diabetes mellitus, and to assess the effectiveness of these interventions on modulating the gut microbiota and improving glucose control.

## Methods

This systematic review was conducted according to a published protocol [26] and the Preferred Reporting Items for Systematic Reviews and Meta-Analyses (PRISMA) guidelines [27].

### Eligibility criteria

Included studies were RCTs or specific arms of non-RCTs that reported on the effectiveness of dietary and physical activity/exercise interventions on modulating the gut microbiota and improving glucose control (i.e. HbA_1c_, fasting blood glucose, 2 h frequently sampled [2 h^fs^] OGTT and/or HOMA-IR or insulin). Participants of interest were adults with type 2 diabetes. Studies reporting on lifestyle interventions in adults with type 1 diabetes were excluded.

It was a requirement that the gut microbiota was measured from stool samples using any form of sequencing technique targeting the 16S ribosomal RNA gene. No standardised criteria defining type 2 diabetes mellitus were specified because the methods for clinical diagnosis vary between studies. However, individuals with type 2 diabetes mellitus had to be clinically diagnosed with their diabetes controlled by diet, oral medication and/or insulin.

Eligible lifestyle interventions included those that targeted diet, physical activity/exercise, use of prebiotics, probiotics or synbiotics, or a combination of these. Studies that included pharmacotherapy, herbal remedies and surgery were excluded. Studies where individuals were already receiving medication as part of their standard care and with no increase in dose during the study were eligible for inclusion.

The primary outcomes of interest were modulation of the gut microbiota, including changes in the relative abundance of bacteria and diversity changes (α and β), and glucose control. The composition of the gut microbiota could also be reported as a secondary outcome. Other secondary outcomes were changes in weight and inflammatory markers (i.e. IL-6, TNF-α, IL-1 receptor antagonist [IL-1RA], C-reactive protein [CRP] and faecal lipocalin-2).

### Search strategy

MEDLINE, EMBASE, Scopus, Web of Science and the Cochrane Library were searched using a combination of MeSH headings and keywords to identify potentially relevant literature (see electronic supplementary material [ESM] Methods). Searches were completed up to 9 February 2017 and were limited to studies published in the English language. Manual searching (i.e. reference lists and citation searching) of studies fulfilling the eligibility criteria was also conducted.

### Selection of studies

Two authors (D. Houghton and T. Hardy) independently screened the titles and abstracts of all the studies generated by the search. Full-text articles retained from the first stage were reassessed independently by the same two authors and assessed by a third independent author (C. Stewart) using a study-selection form. Any disagreements were resolved via discussion with the review team.

### Data extraction

Details of the study population, interventions, comparators and outcomes were captured using a data-extraction form. Data were extracted from all included studies independently by two members of the review team (D. Houghton and T. Hardy). Where applicable, the corresponding authors from the retained studies were contacted via email to request additional information. The Cochrane Collaboration risk of bias tool [28] was used to assess the methodological quality of the included studies and the overall risk of bias (i.e. low, unclear or high). All studies were independently assessed for methodological quality by two authors (D. Houghton and L. Avery).

### Data synthesis

Insufficient data were reported to enable overall effect-size estimates to be calculated using meta-analyses for all outcomes of interest; therefore, the study authors were contacted to request additional outcome data where applicable. This enabled meta-analyses to be conducted for the composition of the gut microbiota (*Bifidobacterium*, *Roseburia* and *Lactobacillus*) and for fasting blood glucose, HbA_1c_, insulin and HOMA-IR levels. Results for other outcomes of interest are presented narratively. Data are expressed as standardised mean difference (SMD) (95% CI) between treatment and control/comparator groups.

## Results

**Fig. 1:**
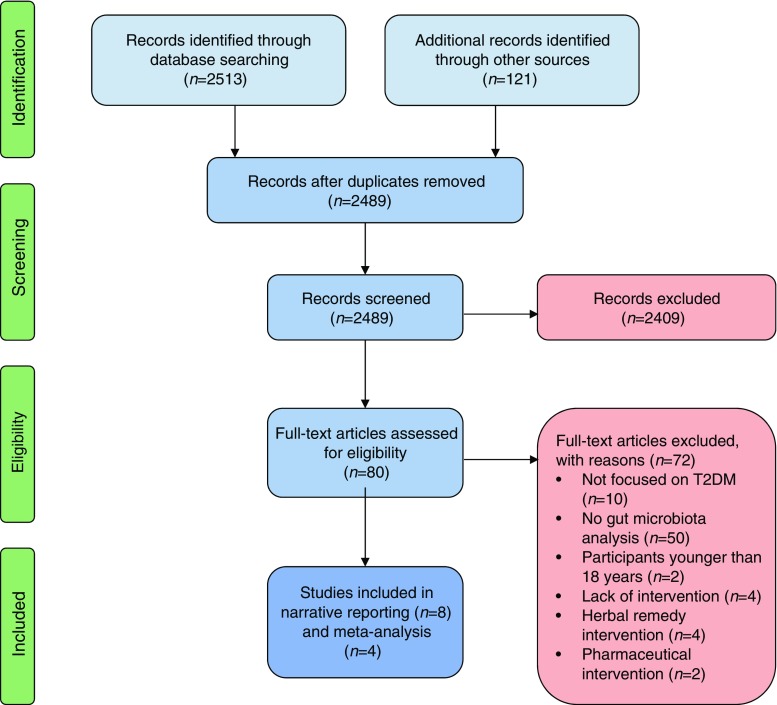
PRISMA diagram presenting the process undertaken to identify eligible studies. T2DM, type 2 diabetes mellitus

The electronic search returned 2513 potentially relevant studies. An additional 121 studies were identified from the end reference lists of the included studies and conference proceedings. Following the removal of duplicates and elimination of ineligible studies, eight studies were retained for review (Fig. 1). All eight studies reported on the effect of dietary interventions (i.e. no studies were retrieved meeting the eligibility criteria that reported on physical activity/exercise alone). These studies were seven RCTs [29–35] and one single-group study [36]. Four of the studies were conducted in Europe (Denmark, Spain, Italy and the UK) and four in Asia (India, Malaysia, Japan and the Republic of Korea). All eight studies used dietary modulation alone (Table 1).

**Table 1:** Summary of included studies

Study ID, country of origin and setting	Details of sample	Details of intervention and study design	Assessment timepoints	Primary outcomes	Secondary outcomes
Sheth et al [35] India Diabetes healthcare clinic	*n* = 35 (treatment arm *n* = 25, control arm *n* = 10) Male: NR Mean age (SD): NR Time since diagnosis: NR Management of T2DM: NR	RCT 1 g synbiotic product (two species of *Lactobacillus* and *Bifidobacterium* each, one species of *Streptococcus* and yeast, and 300 mg oligosaccharide) taken daily with meals	Baseline and 21 days	Glycaemic control: FBG, PP2BS, HbA_1c_	Gut microbiota, SCFAs (butyrate, propionate)
Candela et al [32] Italy University endocrinology and diabetes department	*n* = 40 (additional 13 non-T2DM at baseline; T2DM participants: treatment arm *n* = 21, control arm *n* = 19 included in analysis) Male: 50% Mean age (SD): 58 (16) years Time since diagnosis: NR Management of T2DM: Diet and/or hypoglycaemic drugs for 6 months prior to study	Controlled open-label trial Ma-Pi 2 diet (detailed in [37])	Baseline and 21 days	FBG, PP2BS	HbA_1c_, total cholesterol, LDL-cholesterol and HDL-cholesterol, CRP, TNF-α, IL-6, anthropometrics, gut microbiota
Balfegó et al [29] Spain Hospital clinic, Barcelona	*n* = 35 (treatment arm *n* = 19, control arm *n* = 16) Male: 44% Mean age (SD): 61 (1) years Time since diagnosis: NR Management of T2DM: diet only	Pilot randomised trial Standard T2DM with 100 g sardines on 5 days/week for 6 months	Baseline and 6 months	Glycaemic control: FBG, FBI, HbA_1c_, HOMA-IR	TNF-α, IL-6, IL-8, IL-10, total adiponectin, anthropometrics, nutritional intake
Firouzi et al [33] Malaysia Teaching hospital	*n* = 136 (treatment arm *n* = 68, control arm *n* = 68) Male: 52.2% Mean age (SD): 54 (8) years Time since diagnosis: NR Management of T2DM: oral glucose-lowering drugs	Randomised, double-blind, parallel-group, placebo-controlled trial Probiotics containing 3×10^10^ viable microbial strains twice per day: three strains of *Lactobacillus* (*Lactobacillus acidophilus*, *Lactobacillus casei* and *Lactobacillus lactis*) and three strains of *Bifidobacterium* (*Bifidobacterium bifidum*, *Bifidobacterium longum* and *Bifidobacterium infantis*); contains 10^10^ CFU	Baseline and weeks 6 and 12	Glycaemic control: FBG, FBI, HbA_1c_, HOMA-IR and QUICKI	Anthropometrics, lipid profile, BP, CRP, gut microbiota
Pedersen et al [34] UK General practitioners (Surrey)	*n* = 29 (treatment arm *n* = 14, control arm *n* = 15) Male: 100% Mean age (SD): 57 (2) years Time since diagnosis: NR Management of T2DM: Metformin, *n* = 10 Metformin and gliclazide, *n* = 5 Metformin and sitagliptin, *n* = 3 Metformin, gliclazide and sitagliptin, *n* = 1 Metformin, sitagliptin and thiazolidinedione, *n* = 1 Gliclazide and sitagliptin, *n* = 2 Gliclazide, *n* = 1	Randomised, double-blind, parallel-group, placebo-controlled trial. 5.5 g prebiotic fibre Galacto-oligosaccharide (detailed in [38])	Baseline and 12 weeks	Intestinal permeability, endotoxemia and glycaemic control: FBG, FBI, HbA_1c_, HOMA-IR and C-peptide	Gut microbiota, lipid profile, BP, anthropometrics, CRP, TNF-α, IL-6
Kim et al [36] Republic of Korea Diabetes healthcare clinic	*n* = 6 (*n* = 4 with type 2 diabetes, all included in analysis) Male: 17% Mean age (SD): 60 (12) years Time since diagnosis: NR Management of T2DM: NR	Single-group study Strict vegetarian diet containing 16% protein, 72% carbohydrates (42 g fibre/day) and 12% fat	Baseline and days 3, 5, 7, 14, 21 and 28	FBG	Gut microbiota, 2 h^fs^ OGTT, HbA_1c_, weight, BMI, faecal lipocalin-2, total SCFA, acetate, propionate, butyrate
Sasaki et al [30] Japan Outpatient diabetes clinic	*n* = 66 (treatment arm 300 mg *n* = 23, 900 mg *n* = 22, control arm *n* = 21) Male: 58% Mean age (SD): 62 (9) years Time since diagnosis: NR Management of T2DM: Insulin injection, *n* = 12 Metformin, *n* = 18 Insulin secretagogue, *n* = 38 α-Glucosidase inhibitor, *n* = 31 PPAR-γ antagonist, *n* = 17	RCT Two doses of transglucosidase totalling 300 or 900 mg/day	Baseline and 12 weeks	HbA_1c_	Gut microbiota, FBG, insulin, BMI, nutritional intake
Andreasen et al [31] Denmark General practitioners	*n* = 48 (24 with T2DM; all participants included in analysis: treatment arm *n* = 24, control arm *n* = 24) Male: 100% Mean age (SD): 62 (8.9) years Time since diagnosis: NR Management of T2DM: Metformin, *n* = 9 Sulfonylurea, *n* = 3 Glitazones, *n* = 1, Statins, *n* = 5	RCT 1 g probiotic capsule/day containing 10^10^ CFU *Lactobacillus acidophilus* NCFM	Baseline and 4 weeks	Gut microbiota	FBG, HbA_1c_, BMI^,^ 2 h^fs^ OGTT, HOMA-IR, FBI, TNF-α, IL-6, IL-1RA, CRP

CFU, colony-forming units; FBG, fasting blood glucose; FBI, fasting blood insulin; NR, not reported; PP2BS, postprandial blood glucose; T2DM, type 2 diabetes mellitus

### Characteristics of participants with type 2 diabetes

The total sample size across the included studies was 395, including at least 225 men and 135 women; one study failed to report the sex of participants (*n* = 35) [35]. Participants were aged 33–77 years (58 ± 4 years [mean ± SD]) with a BMI range of 25–45 kg/m^2^. Six studies recruited only individuals diagnosed with type 2 diabetes mellitus [29, 30, 32–35]. One study compared participants with type 2 diabetes mellitus and control participants at baseline, prior to the introduction of any intervention, however, these 13 participants were not included in the analyses reported here [32]. Two studies [31, 36] reported on a sample including individuals with and without type 2 diabetes mellitus (Table 1). Only one study reported the ethnicity of participants and none of the included studies reported the time since diagnosis of type 2 diabetes mellitus. Five studies reported treatment regimens: insulin injection (*n* = 11), insulin secretagogue (*n* = 34), metformin (*n* = 52), sulfonylurea (*n* = 12), α-glucosidase inhibitor (*n* = 41), sitagliptin (*n* = 7), thiazolidinedione (*n* = 1), peroxisome proliferator-activated receptor γ (PPAR-γ) antagonist (*n* = 15) or statins (*n* = 5) [30, 31, 34]. One study recruited individuals who managed their type 2 diabetes mellitus through diet only [29], and two studies used eligibility criteria that restricted the use of insulin but allowed hypoglycaemic drugs and dietary interventions, but provided no specific details [32, 33]. Two studies [35, 36] did not report any treatment regimens (Table 1).

### Intervention characteristics

The duration of interventions ranged from 21 days to 6 months. All included studies involved some form of dietary intervention, including dietary manipulation and/or supplements. One RCT supplemented participants with a synbiotic [35], one single-group study instructed participants to follow a strict vegetarian diet [36], one RCT [32] instructed participants to follow a strict Ma-Pi diet [37] and a further RCT asked participants to follow a diet recommended for type 2 diabetes mellitus incorporating an increased intake of sardines [29]. The last two of these studies included information on nutrient intake, although Balfegó et al [29] did not provide any information on what constituted the type 2 diabetic diet. Two RCTs provided participants with probiotics [31, 33], but only one recorded nutrient intake and provided guidelines on dietary intake based on type 2 diabetes mellitus guidelines [33]. One RCT provided a prebiotic, detailed in Vulevic et al [38], with no dietary advice, but recorded nutrient intake [34]; and one RCT provided participants with a digestive supplement and recorded nutrient intake at baseline [30].

All eight studies reported on glucose control, including HbA_1c_, fasting blood glucose, 2 h^fs^ OGTT, HOMA-IR or insulin, or a combination of these, and the gut microbiota (using various techniques; ESM Table 1).

### Methodological quality assessment

All included studies were assessed for methodological quality using the Cochrane Collaboration risk of bias tool (Table 2) [28]. Seven studies explicitly reported their hypotheses, objectives, statistical testing procedures and main findings. Four studies reported a power calculation including details of whether sample size was obtained [30, 31, 33, 34]. Only one study retained the sample size at follow-up [31]. One study provided a power calculation based on changes in TNF-α upon request [31]. However, it is unclear from the information reported in the article whether this was the primary outcome, making it difficult to establish whether the sample size was adequate. All but one study [35] reported attrition rates, and one study reported using an intention-to-treat analysis [33].

**Table 2:** Methodological quality assessment and grading of studies

Study	Power calculation			Attrition rate	Intention to treat	Methodological quality assessment^a^						
	Reported (yes/no)	Sample size achieved	Sample size retained			A	B	C	D	E	F	Risk of bias within study
Sheth et al [35]	No	NA	NA	NR	NR	Unclear	No	No	Unclear	Yes	Yes	Unclear
Candela et al [32]	No	NA	NA	*n* = 0 (0%)	NR	Unclear	No	No	Yes	Yes	Yes	Low
Balfegó et al [29]	No	NA	NA	*n* = 3 (9%)	NR	Yes	No	No	Yes	Yes	Yes	Low
Firouzi et al [33]	Yes	Yes	No	*n* = 35 (26%)	Yes	Yes	Yes	Yes	Yes	Yes	Yes	Low
Pedersen et al [34]	Yes	Yes	No	*n* = 2 (6%)	NR	Yes	No	No	Yes	Yes	Yes	Low
Kim et al [36]	No	NA	NA	*n* = 0 (0%)	NR	NA	NA	NA	Yes	NA	Yes	Low
Sasaki et al [30]	Unclear^b^	Yes	No	*n* = 6 (9.1%)	NR	Yes	Yes	Yes	Yes	NA	Yes	Low
Andreasen et al [31]	Yes^c^	Yes	Yes	*n* = 3 (6.5%)	NR	Unclear	Unclear	Yes	Yes	No	Yes	Low

^a^A, adequate sequence generation; B, allocation concealment; C, blinding/masking; D, incomplete outcome data addressed; E, free of selective outcome reporting; F, study free of other problems

^b^The authors reported a power calculation in a previous study, but not in the study included in the current review

^c^The authors provided a power calculation upon request for changes in TNF-α. It was unclear whether change in TNF-α was the primary outcome of the study

NA, not applicable; NR, not reported

Four studies provided enough information to confirm the use of adequate sequence generation [29, 30, 33, 34], two studies provided sufficient detail on the methods used to conceal allocation sequences [30, 33] and three studies provided explicit detail on blinding of the research team [30, 31, 33]. Seven studies provided detail on outcome assessors [29–34, 36] and five studies provided sufficient detail to establish the likely absence of selective outcome reporting [29, 32–35]. Overall, seven of the studies were considered to be low risk of bias [29–34, 36] and the risk of bias of one [35] was unclear (Table 2).

### Effects of lifestyle modulation on gut microbiota

Additional data on the gut microbiota were requested from all authors of the included studies. Two studies comparing supplementation with a prebiotic vs control for 12 weeks [34] and the Ma-Pi diet vs a control diet for 21 days [32] provided sufficient data for meta-analyses to be conducted. Changes in the relative abundance of bacteria in the genera *Bifidobacterium* (SMD 1.29% [95% CI −4.45, 7.03], *I*^2^ = 33%), *Roseburia* (SMD −0.85% [95% CI −2.91, 1.21], *I*^2^ = 79%) and *Lactobacillus* (SMD 0.04% [95% CI −0.01, 0.09], *I*^2^ = 0%) (ESM Fig. 1) were reported, however, these changes were not significantly different when comparing dietary interventions with controls.

Candela et al [32] compared a fibre-rich macrobiotic diet with a control diet. The authors reported a significant change in weighted UniFrac following receipt of the intervention. Levels of *Faecalibacterium* were significantly negatively correlated with fasting blood glucose; *Akkermansia* and *Bacteroides* both showed a positive significant relationship with LDL-cholesterol; and *Ruminococcus* was significantly positively correlated with fasting blood glucose. Significant increases in the relative abundance of Peptostreptococcaceae and Leuconostocaceae were also reported, and both of these *genera* were positively correlated with dietary components (fermented products).

Balfegó et al [29] compared a type 2 diabetes diet, one enriched with 100 g of sardines and one without. The authors reported a decrease in Firmicutes and an increase in *Escherichia coli* in both groups between baseline and study completion. In the sardine enriched group there was also a decrease in the Firmicutes:Bacteroidetes ratio and an increase in Bacteroides-*Prevotella* when compared with baseline. Kim et al [36] reported a significant increase in the relative abundance of Bacteroidetes and a correlation between weighted and unweighted UniFrac and a strict vegetarian diet. However, these analyses involved the whole sample of participants, including those without a confirmed diagnosis of type 2 diabetes. Two RCTs [31, 33] supplemented participants with probiotics and reported significant changes in bacterial composition: Andreasen et al [31] reported a significant increase in the presence of *Lactobacillus acidophilus* from near non-detectable levels to 6.4 colony-forming units; and similarly, Firouzi et al [33] reported significant increases of 4.5- and twofold in *Bifidobacterium* and *Lactobacillus* spp., respectively.

Pedersen et al [34] conducted an RCT in which participants were supplemented with a prebiotic compared with placebo. No between-group differences were shown; however, an increase in α diversity was reported within the prebiotic group. Furthermore, correlations between bowel permeability, metabolic profile, inflammatory markers and bacteria were reported (ESM Table 2). Sheth et al [35] supplemented participants with a synbiotic (two species of *Lactobacillus* and *Bifidobacterium* each, one species of *Streptococcus* and yeast, and 300 mg oligosaccharide), although the dietary intake provided alongside the supplements was unclear. Increases in both *Lactobacillus* and *Bifidobacterium* were reported following the intervention. Sasaki et al [30] reported significant changes in the Firmicutes:Bacteroidetes ratio between baseline and 12 weeks following supplementation with 300 and 900 mg/day of transglucosidase.

### Effects of dietary intervention modulation on glucose control

Four studies comparing dietary interventions including prebiotics, probiotics and Ma-Pi diet vs controlled diets for a duration of between 21 to 84 days reported or provided sufficient data to enable meta-analyses of fasting blood glucose, HbA_1c_, fasting insulin and HOMA-IR (ESM Fig. 2) [30, 32–34]. Reductions were shown in all glucose control variables. HbA_1c_ was significantly reduced (standardised mean difference [SMD] −2.31 mmol/mol [95% CI −2.76, −1.85] [0.21%; 95% CI −0.26, −0.16]; *I*^*2*^ = 0%, *p* < 0.01), however, fasting blood glucose (SMD −0.25 mmol/l [95% CI −0.85, 0.35], *I*^*2*^ = 87%, *p* > 0.05), fasting insulin (SMD −1.82 pmol/l [95% CI −7.23, 3.60], *I*^*2*^ = 54%, *p* > 0.05) and HOMA-IR (SMD −0.15 [95% CI −0.63, 0.32], *I*^*2*^ = 69%, *p* > 0.05) were not significantly reduced when comparing dietary interventions with comparator groups.

All eight studies reported on glucose control; however, only four provided sufficient data to calculate overall effect sizes [30, 32–34] (ESM Fig. 2). Kim et al [36] and Sasaki et al [30] reported positive changes in fasting blood glucose, 2 h^fs^ OGTT, fasting insulin and HbA_1c_, although these were not statistically significant. Andreasen et al [31] reported baseline fasting blood glucose, HbA_1c_, 2 h^fs^ OGTT and HOMA-IR, but did not report data for these variables at the 4 week follow-up point. A significant between-group improvement in insulin sensitivity was reported, but post-hoc *t* tests revealed no significant within-group changes. Unfortunately, analyses were not reported for participants with type 2 diabetes mellitus only, making it difficult to extrapolate the effects of the intervention.

Two RCTs showed significant improvements in glucose control [29, 32]. Candela et al [32] reported reductions in fasting blood glucose (−2.3 vs −1.9 mmol/l), postprandial blood glucose (−4.0 vs −4.3 mmol/l), HbA_1c_ (−5.5 vs −2.2 mmol/mol [−0.5% vs −0.2%] and HOMA-IR (−1.9 vs −1.5). Balfegó et al [29] reported significant reductions in fasting insulin (−35% vs −23%) and HOMA-IR (−49% vs −22%) for treatment and comparator groups, respectively. Three RCTs supplemented participants with a prebiotic, probiotic or synbiotic. Firouzi et al [33] reported significant reductions in insulin at 6 and 12 weeks (−16 and −20 pmol/l, respectively) and HbA_1c_ at 12 weeks (−1.1 mmol/mol [−0.1%]) between the probiotic and control group. Sheth et al [35] reported reductions in fasting blood glucose, postprandial blood glucose and HbA_1c_; however, it is unclear from the reported findings whether differences between groups were assessed, as data were not provided. Pedersen et al [34] reported no statistical improvements in glucose control following supplementation with prebiotics.

### Effects of dietary intervention modulation on inflammation

Four studies reported no significant differences in the inflammatory markers TNF-α, IL-6, IL-1RA or CRP between groups [29, 31, 33, 34]. Kim et al [36] reported significant reductions in faecal lipocalin 2; however, these analyses included participants without type 2 diabetes mellitus, making it difficult to extrapolate results for the subgroup of participants with type 2 diabetes. One RCT reported significant reductions in TNF-α, IL-6 and CRP following 21 days consuming the Ma-Pi 2 diet [32].

### Effects of dietary intervention on short-chain fatty acids

Kim et al [36] reported a significant increase in butyrate and a reduction in total short-chain fatty acids (SCFAs), acetate, propionate and butyrate concentrations. However, these analyses were conducted on all individuals recruited, including those without a confirmed diagnosis of type 2 diabetes. Sheth et al [35] also reported increased concentrations in butyrate and propionate, although it is unclear what statistical analyses were conducted.

### Effects of dietary intervention on anthropometrics

Seven studies reported BMI at baseline [29–34, 36] and six of these studies reported BMI postintervention [29, 30, 32–34, 36]. Five studies reported no significant changes in anthropometrics, including weight, BMI and hip and waist circumferences [29, 30, 32–34]. Kim et al [36] provided BMI data upon request showing a postintervention reduction; however, it is unclear from the published article or the additional information provided whether this was statistically significant.

### Changes in nutrition

Four studies recorded nutritional intake at baseline and the postintervention follow-up, including energy intake and the consumption of fat, protein, carbohydrates and fibre. Although Candela et al [32] reported dietary intake, no statistical analyses were reported. Firouzi et al [33] reported a statistically significant 9% reduction in fat intake and Balfegó et al [29] reported an 11% reduction in energy intake. Pedersen et al [34] reported a statistically significant 1.1% increase in protein intake in the control group compared with the prebiotic group.

## Discussion

This systematic review aimed to assess the effectiveness of lifestyle interventions targeting diet and/or physical activity/exercise for modulating the gut microbiota and improving glucose control in adults with type 2 diabetes. Previous reviews have reported the effects of lifestyle interventions on their ability to improve glucose control in adults with type 2 diabetes [39–42]. To the best of our knowledge, however, there have been no published reviews on the effectiveness of lifestyle interventions targeting the gut microbiota in combination with changes in glucose control in adults with type 2 diabetes. Although no eligible studies were retrieved reporting on the effect of physical activity/exercise on the gut microbiota and metabolic health, the evidence generated by this review demonstrates that dietary interventions can modulate the gut microbiota while improving glucose control.

Advances in microbiome research have revealed the importance of individual variability in the composition of the gut microbiota in supporting health and contributing to disease [6]. One key environmental factor that shapes the gut microbiota composition is diet, influencing gut transit time, pH and macronutrient ingestion [43]. All of the studies reviewed here reported significant changes in the gut microbiota composition following dietary intervention [29–36], supporting the findings of previously published studies in children and adults [44, 45]. The current review found that changes in metabolic health were closely related to significant changes in gut microbiota composition, including changes at various taxa levels (e.g. phylum, family, genus, species and Firmicutes:Bacteroidetes ratio).

Meta-analysis showed no significant changes in the relative abundance of three potentially healthy-gut-promoting bacteria at the genus level: *Bifidobacterium*, *Roseburia* and *Lactobacillus*. A potential explanation is the small sample size of the intervention groups (*n* = 21 and *n* = 14) and short duration (3 and 12 weeks) of the included trials, the content/nature of the dietary intervention (Ma-Pi 2 diet and prebiotic) and the small number of studies included in the meta-analysis (*n* = 2) [32, 34], respectively. In contrast to the outcome of the meta-analysis, all of the studies reviewed did report statistically significant changes in different gut microbiota variables. For example, significant changes were reported in the relative abundances at the phylum (Firmicutes, Bacteroidetes), family (Peptostreptococcaceae and Leuconostocaceae) genus (*Prevotella*, *Bifidobacterium* and *Lactobacillus*) and species (*L. acidophilus*) level, and in α (Shannon and inverse Simpson) and β (weighted and unweighted UniFrac) diversity matrices. Furthermore, significant correlations were reported between various bacteria and metabolic variables such as fasting blood glucose and LDL-cholesterol. These data provide support for the ability of dietary intervention to modulate the gut microbiota; however, the gut microbiota field is still relatively young and our understanding of what these changes mean is limited. This issue is further complicated by observations being made at the bacterial level and not taking into account functionality, as reported in this review.

A potential issue identified was variability among participants within the gut microbiota studies reviewed. Furthermore, to observe a genuine effect of an intervention in this field requires an adequate sample size to account for the substantial variability both within and between studies reporting on the gut microbiota, and strictly controlled intervention studies that adopt consistent methods and interventions to allow comparisons to be made. The heterogeneity of the results highlighted could be explained by these issues.

The mechanisms linking changes in bacterial composition to metabolic dysfunction have not been confirmed. Potential mechanisms include: (1) altered levels of glucagon-like peptide-1 and -2 [15]; (2) increased lipopolysaccharides; (3) inflammation [18]; (4) reduced SCFAs and appetite [46]; and (5) increased energy extraction from digesta [12]. Although not exhaustive, this list highlights the potential contributions of the gut microbiota to type 2 diabetes; however, the pathophysiological processes remain unknown. This is primarily because the gut microbiome field is still in its infancy, although we will undoubtedly learn more about the contribution of the gut microbiota towards type 2 diabetes mellitus in this growing field of research. However, this review does support the importance of dietary change and its ability to modulate the gut microbiota in an attempt to maintain gut homeostasis and improve glucose control, as previously shown [13]. Targeting the gut microbiota and/or using the information generated from gut microbiome studies could offer an alternative therapeutic approach that takes into account the substantial variability observed among individuals with type 2 diabetes mellitus.

In addition to dietary change, previous research has shown that exercise impacts positively on health outcomes, including immune function, and has anti-inflammatory effects [22, 47]. However, the search conducted for this systematic review did not identify any studies reporting on the effects of physical activity/exercise on the gut microbiota and glucose control that met the eligibility criteria. This highlights a gap in the evidence base and thus a need for well-conducted studies in this area. At the time of the search, the studies by Clarke et al [22] and Shukla et al [48] were the only ones to have investigated the effects of exercise on the gut microbiota in humans, reporting positive and negative outcomes, respectively, questioning its use in clinical practice.

A body of evidence from clinical studies [49] and preclinical models [50, 51] demonstrates that exercise reduces inflammation, upregulates glucagon-like peptide-1 secretion [52] and modulates the gut microbiota. Furthermore, interventions targeting physical activity/exercise have also been shown to improve glucose control [41], body composition and liver fat in humans with fatty liver disease [53] and type 2 diabetes mellitus [54], irrespective of weight loss. The exact mechanisms behind how physical activity/exercise modulates the gut microbiota and risk factors for type 2 diabetes mellitus in combination remain unknown. Potential mechanisms include altered SCFAs, cholesterol metabolism, substrate for bacterial growth and gastrointestinal tract transit time; however, this review highlights a lack of research and therefore understanding in this area, which hinders its clinical use at present.

This systematic review has provided evidence to demonstrate that diet has an important role in modulating the gut microbiota, which has been linked with disease pathophysiology. This has important clinical implications and further supports the importance of targeting the gut microbiota in lifestyle interventions as a therapeutic pathway in clinical practice.

Strengths of this systematic review include the transferability of the findings to clinical environments (the included studies were conducted in primary- and secondary-care settings) and the clinical group studied (i.e. people with type 2 diabetes mellitus), demonstrating potential clinical use. Despite the small numbers of studies reviewed (*n* = 8), encouragingly all scored high in terms of methodological quality, suggesting that the conclusions can be considered reliable. However, it should be noted that the studies included were small in terms of sample size (*n* = 395) and were of short duration, and samples consisted of a disproportionately small number of women, which could limit generalisability. Although all of the studies included incorporated dietary modification, including specific diets and/or some form of supplementation, these were heterogeneous in nature (i.e. the diets used differed among studies in terms of the dietary approach and nutritional content). Dietary supplementation was commonly used, and generic probiotics, prebiotics and synbiotics were provided. Finally, various methods of sequencing 16S ribosomal RNA (culture methods vs next-generation sequencing) and reporting results meant that levels of heterogeneity arising from the meta-analyses conducted were often high, and make an overall understanding difficult.

### Conclusion/future directions

Lifestyle intervention holds potential for improving the gut microbiota and glucose control. To truly use the potential of the gut microbiota and its implications for disease pathophysiology and health, future studies should consider using an appropriate intervention duration and sample size, and conducting long-term follow-up to assess whether changes in the gut microbiota and glucose control are maintained. Lifestyle behaviour change through a combination of diet and/or physical activity/exercise has been shown to have a significant beneficial impact upon glucose control [55]. However, the role that the gut microbiota plays in this process remains unclear. Another unanswered question is why some individuals respond to interventions more profoundly than others. To date, studies have focused predominantly on preclinical models, limiting their transferability into humans for use as therapeutic interventions. This review highlights the need for further well-conducted human intervention studies. As the pathology of type 2 diabetes mellitus advances and our understanding of the gut microbiota increases, there is significant potential to determine the importance of specific bacteria and their function in a given bacterial community. This will, in turn, lay the foundation for translating preclinical data into clinical practice by integrating multiple techniques and the characteristics of individuals into a systems biology approach to provide personalised lifestyle interventions.

### Contribution statement

DH, TH, CS, LE, CPD, LA and MIT conceived the review protocol. LA and MIT supervised the review. DH, TH, CS, LE and LA developed the search strategy. DH and LE performed the electronic searches. DH, TH and CS screened titles and abstracts, and evaluated the eligibility of full-text articles. DH and LA assessed the included studies for methodological quality and the overall risk of bias. All authors provided input to the development of the methods and interpretation of the results. DH and LA drafted the manuscript and all authors commented upon the manuscript for important intellectual content. All authors approved and gave their permission for the final version to be published. DH and LA are responsible for the integrity of the work as a whole.

## Electronic supplementary material

ESM (PDF 550 kb)

## Data Availability

All data relating to this systematic review are available upon request from the first author (D. Houghton).

## Abbreviations

CRP: C-reactive protein
2 h^fs^ OGTT: 2 h frequently sampled OGTT
IL-1RA: IL-1 receptor antagonist
PPAR-γ: Peroxisome proliferator-activated receptor γ
SCFA: Short-chain fatty acid
SMD: Standardised mean difference
