# Supplementary material for: Systematic review assessing the effectiveness of dietary intervention on gut microbiota in adults with type 2 diabetes
Source: Diabetologia. 2018 May 12;61(8):1700–11. doi: 10.1007/s00125-018-4632-0 (PMC6061157; doi:10.1007/s00125-018-4632-0)
Supplement: Supplementary file 1 — (PDF 550 kb) [file 125_2018_4632_MOESM1_ESM.pdf]

ESM Methods: Search Strategy

The following databases were searched: Medline, EMBASE, Scopus, Web of Science, Cochrane Database of Systematic Reviews, Cochrane Controlled Trials Register by a medical librarian and confirmed by DH. Manual searches of citations and references lists of studies fulfilling the eligibility criteria was also undertaken by DH, TH and CS. The databases were searched using a combination of MeSH headings and keywords to identify potentially relevant studies. The final full list of terms used for searching Ovid Medline can be seen below. This includes search strings suggested by the Cochrane Collaboration to effectively identify RCTs and research in humans. This search strategy was then adapted as closely as possible to take into account differing indexing terms, search functionality and suggested search strings for the other databases. These additional search strategies are available on request.

Database: Ovid MEDLINE(R)
Search Strategy:

1   "gut microbiota".mp.
2   exp Microbiota/
3   microbiome.mp.
4   2 or 3
5   gut.mp.
6   faecal.mp.
7   5 or 6
8   4 and 7
9   "gut flora".mp.
10   Dysbiosis/
11   eubiosis.mp.
12   exp Endotoxins/
13   exp bacteria/
14   1 or 8 or 9 or 10 or 11 or 12 or 13
15   exp Motor Activity/
16   "physical activity".mp.
17   exp Exercise/
18   exp Diet/
19   diet.mp.

20 nutrition.mp.
21 exp Life Style/
22 lifestyle.mp.
23 "life style".mp.
24 prebiotics/
25 probiotics/
26 or/15-25.
27 "insulin insensitivity".mp.
28 exp Insulin Resistance/
29 "insulin resistance".mp.
30 exp Inflammation/
31 inflammation.mp.
32 exp Diabetes Mellitus, Type 2/
33 t2dm.mp.
34 glucose intolerance/
35 27 or 28 or 29 or 30 or 31 or 32 or 33 or 34 or 35 or 36 or 37 or 38 or 39 or 40 or 41 or 42 or 43 or 44 or 45
36 randomized controlled trial.pt.
37 controlled clinical trial.pt.
38 randomized.ab.
39 trial.ti.
40 placebo.ab.
41 clinical trials as topic.sh.
42 randomly.ab.
43 exp animals/ not humans.sh.
44 14 and 26

ESM Table 1: Microbiota analysis

Study ID	DNA Extraction Method	Bacteria Sequenced	Sequencing Technique	Analysis Methods
Kim et al. [36]	Not Specified	16S rRNA gene,<br>regions V1-V2	454-pyrosequencing<br>(GS FLX System)	Not Specified
Sasaki et al. [30]	4M Guanidinium Thiocyanate<br>100mM Tris-HCL<br>40mM Ethylenediaminetetraacetic acid	16S rRNA gene, region<br>not specified	Terminal Restriction<br>Fragment Length	Gene Mapper Software<br>GeneMaths
Andreasson et al. [31]	QIAamp DNA Stool Mini Kit	Lactobacillus Acidophilus	PCR and qPCR	N/A
Sheth et al. [35]	QIAgen Kit	16S rRNA gene<br>regions V6-V8	PCR and qPCR	Not Specified
Pedersen et al. [34]	QIAsymphony automated<br>extraction platform (Qiagen)	16S rRNA gene<br>regions V4-V5	GS FLX<br>Titanium (Roche<br>Diagnostics)	QIIME
Candela et al. [32]	DNeasy Blood and Tissue Kit<br>QIAgen	16S rRNA gene<br>regions V3-V4	Illumin MiSeq	PANDAseq<br>QIIME
Balfego et al. [29]	PSP Spin Stool DNA Kit (Stratec)	*Faecalibacterium*<br>*Prausnitzii,*<br>*Escherichia coli,*<br>*Eubacterium rectale,*<br>*Clostridium coccoides,*<br>*Bacteroides,*<br>*Prevotella,*<br>Firmicutes,<br>Firmicutes/Bacteroidetes ratio	PCR and qPCR	N/A
Firouzi et al. [33]	Not specified	*Lactobacillus spp*<br>*Bifidobacterium spp*	Media Culturing	N/A

ESM Table 2: Correlations between changes in primary and secondary outcomes

	Positive Correlation		Negative Correlations	
Pedersen et al. [34]	Large Bowel Permeability at 6-24 hours	*Flavonifractor*<br>*Verrucomicrobia*<br>*Euryarchaeota*<br>*Mathanobacteria*<br>*Rikenellaceae*<br>*Clostridiales*<br>*Alistipes*<br>*Shigella*	sCD14	*Verrucomicrobi*,<br>*Unclassified*<br>Erysipelotrichales,<br>*Verrucomicrobiales*,<br>*Verrucomicrobiacea*,<br>*Lactobacillaceae*,<br>*Erysipelotrichaceae*
	Small Intestine and Total Intestine permeability at 0-6 and 0-24 hours	Enterobacteriaceae		
	Glucose totalAUC	*Actinobacteria*<br>*Bifidobacterium*	Glucose totalAUC	*Veillonellacea*,<br>*Clostridium* 279 cluster XVIII
	Fasting Blood Glucose	Unclassified Enterobacteriaceae 280		
	Insulin Sensitivity	Unclassified Enterobacteriaceae 280		
	hsCRP	Unclassified Enterobacteriaceae 280		
	Waist Circumference	Unclassified Enterobacteriaceae 280		
	IL-6	*Actinobacteria*<br>*Bifidobacterium*	IL-6	*Veillonellaceae*<br>*Dialister*
	TNF-a	Firmicutes		
Candela et al. [32]	Total and LDL-Cholesterol	*Bacteroides*		

		Akkermansia		
	Fasting blood glucose	Lachnospiraceae<br>*Ruminococcus*	Fasting blood glucose	*Faecalibacterium*
	Dietary Components (eg. whole grain, vegetables, sauces, herbs, seeds, seaweeds and fermented products)	Peptostreptococcaceae<br>Leuconostocaceae		

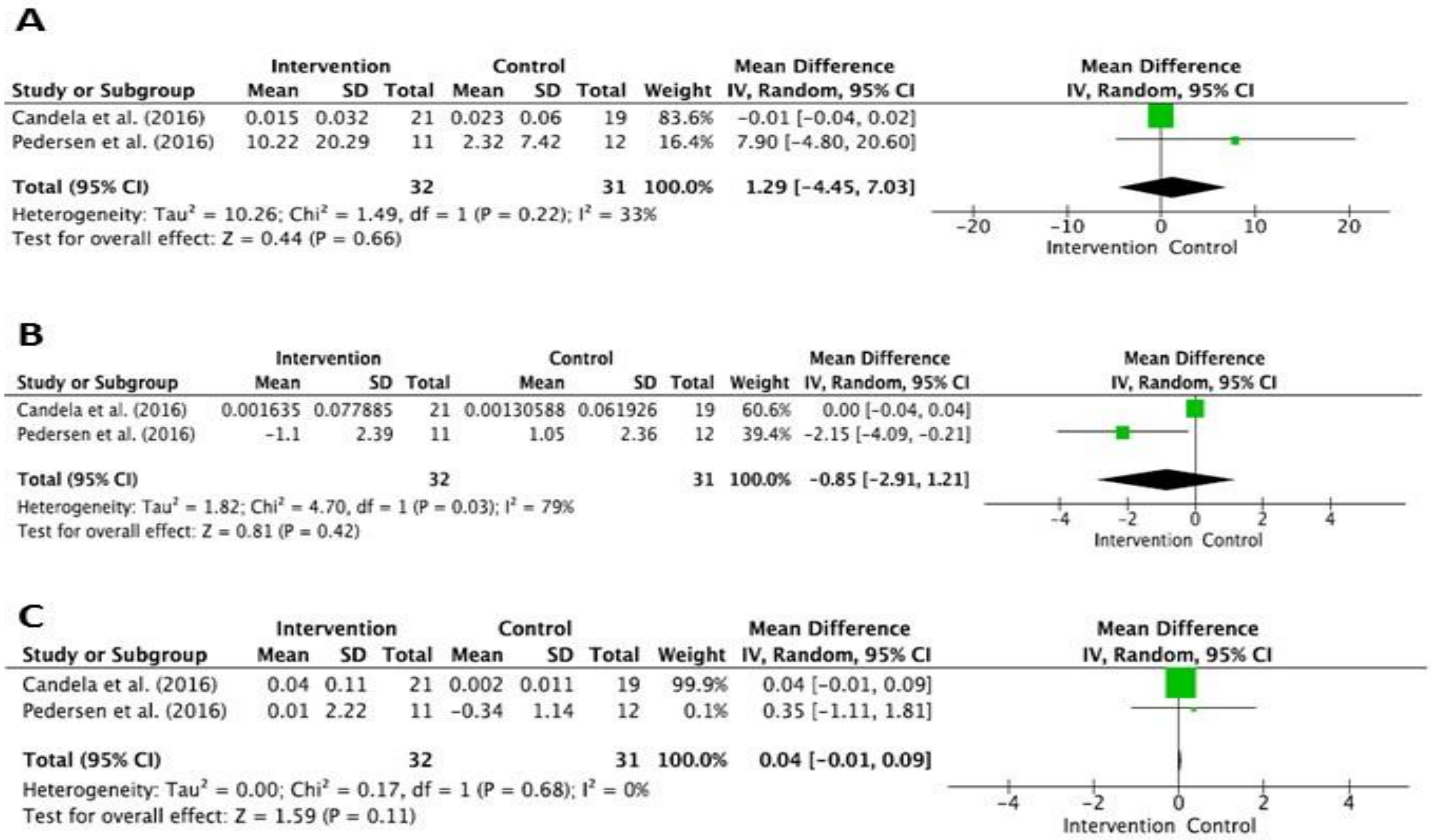

ESM Figure 1: Forest plots reporting the effects of Ma-Pi diet and prebiotic supplementation on *genus* bacteria *Bifidobacterium* (a), *Roseburia* (b) and *Lactobacillus* (c).

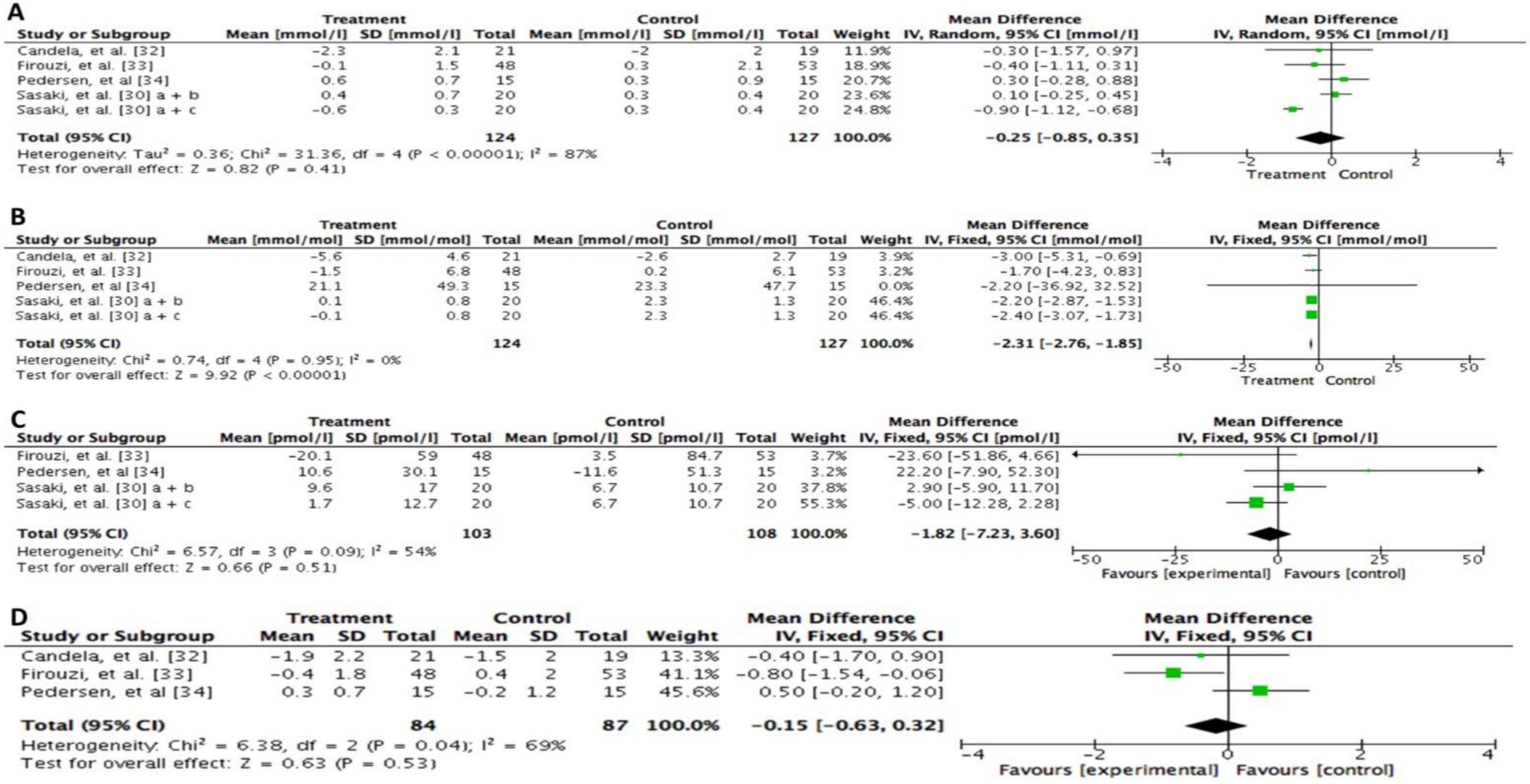

A

Study or Subgroup	Treatment Mean [mmol/l]	Treatment SD [mmol/l]	Treatment Total	Control Mean [mmol/l]	Control SD [mmol/l]	Control Total	Weight	Mean Difference IV, Random, 95% CI [mmol/l]
Candela, et al. [32]	-2.3	2.1	21	-2	2	19	11.9%	-0.30 [-1.57, 0.97]
Firouzi, et al. [33]	-0.1	1.5	48	0.3	2.1	53	18.9%	-0.40 [-1.11, 0.31]
Pedersen, et al [34]	0.6	0.7	15	0.3	0.9	15	20.7%	0.30 [-0.28, 0.88]
Sasaki, et al. [30] a + b	0.4	0.7	20	0.3	0.4	20	23.6%	0.10 [-0.25, 0.45]
Sasaki, et al. [30] a + c	-0.6	0.3	20	0.3	0.4	20	24.8%	-0.90 [-1.12, -0.68]
**Total (95% CI)**			**124**			**127**	**100.0%**	**-0.25 [-0.85, 0.35]**

Heterogeneity: $Tau^2 = 0.36$; $Chi^2 = 31.36$, df = 4 ($P < 0.00001$); $I^2 = 87\%$
Test for overall effect: Z = 0.82 (P = 0.41)

B

Study or Subgroup	Treatment Mean [mmol/mol]	Treatment SD [mmol/mol]	Treatment Total	Control Mean [mmol/mol]	Control SD [mmol/mol]	Control Total	Weight	Mean Difference IV, Fixed, 95% CI [mmol/mol]
Candela, et al. [32]	-5.6	4.6	21	-2.6	2.7	19	3.9%	-3.00 [-5.31, -0.69]
Firouzi, et al. [33]	-1.5	6.8	48	0.2	6.1	53	3.2%	-1.70 [-4.23, 0.83]
Pedersen, et al [34]	21.1	49.3	15	23.3	47.7	15	0.0%	-2.20 [-36.92, 32.52]
Sasaki, et al. [30] a + b	0.1	0.8	20	2.3	1.3	20	46.4%	-2.20 [-2.87, -1.53]
Sasaki, et al. [30] a + c	-0.1	0.8	20	2.3	1.3	20	46.4%	-2.40 [-3.07, -1.73]
**Total (95% CI)**			**124**			**127**	**100.0%**	**-2.31 [-2.76, -1.85]**

Heterogeneity: $Chi^2 = 0.74$, df = 4 ($P = 0.95$); $I^2 = 0\%$
Test for overall effect: Z = 9.92 ($P < 0.00001$)

C

Study or Subgroup	Treatment Mean [pmol/l]	Treatment SD [pmol/l]	Treatment Total	Control Mean [pmol/l]	Control SD [pmol/l]	Control Total	Weight	Mean Difference IV, Fixed, 95% CI [pmol/l]
Firouzi, et al. [33]	-20.1	59	48	3.5	84.7	53	3.7%	-23.60 [-51.86, 4.66]
Pedersen, et al [34]	10.6	30.1	15	-11.6	51.3	15	3.2%	22.20 [-7.90, 52.30]
Sasaki, et al. [30] a + b	9.6	17	20	6.7	10.7	20	37.8%	2.90 [-5.90, 11.70]
Sasaki, et al. [30] a + c	1.7	12.7	20	6.7	10.7	20	55.3%	-5.00 [-12.28, 2.28]
**Total (95% CI)**			**103**			**108**	**100.0%**	**-1.82 [-7.23, 3.60]**

Heterogeneity: $Chi^2 = 6.57$, df = 3 ($P = 0.09$); $I^2 = 54\%$
Test for overall effect: Z = 0.66 (P = 0.51)

D

Study or Subgroup	Treatment Mean	Treatment SD	Treatment Total	Control Mean	Control SD	Control Total	Weight	Mean Difference IV, Fixed, 95% CI
Candela, et al. [32]	-1.9	2.2	21	-1.5	2	19	13.3%	-0.40 [-1.70, 0.90]
Firouzi, et al. [33]	-0.4	1.8	48	0.4	2	53	41.1%	-0.80 [-1.54, -0.06]
Pedersen, et al [34]	0.3	0.7	15	-0.2	1.2	15	45.6%	0.50 [-0.20, 1.20]
**Total (95% CI)**			**84**			**87**	**100.0%**	**-0.15 [-0.63, 0.32]**

Heterogeneity: $Chi^2 = 6.38$, df = 2 ($P = 0.04$); $I^2 = 69\%$
Test for overall effect: Z = 0.63 (P = 0.53)

ESM Figure 2: Forest plots reporting the effects of dietary interventions on glucose control; fasting blood glucose (a), HbA1c (b), insulin (c) and HOMA-IR (d)
